# Case report: Characteristics and nature of primary cardiac synovial sarcoma

**DOI:** 10.3389/fonc.2024.1361414

**Published:** 2024-04-03

**Authors:** Tomonori Kawasaki, Tomomi Nakajima, Tomoaki Torigoe, Kojiro Onohara, Kentaro Ishii, Satoshi Kanno, Chisako Muramatsu, Rikito Tatsuno, Takahiro Jubashi, Jiro Ichikawa

**Affiliations:** ^1^ Department of Pathology, Saitama Medical University International Medical Center, Saitama, Japan; ^2^ Department of Cardiovascular Surgery, Saitama Medical University International Medical Center, Saitama, Japan; ^3^ Department of Orthopedic Oncology & Surgery, Saitama Medical University International Medical Center, Saitama, Japan; ^4^ Department of Radiology, Interdisciplinary Graduate School of Medicine, University of Yamanashi, Chuo, Yamanashi, Japan; ^5^ Faculty of Data Science, Shiga University, Hikone, Japan; ^6^ Department of Orthopedic Surgery, Interdisciplinary Graduate School of Medicine, University of Yamanashi, Yamanashi, Japan

**Keywords:** ynovial sarcoma, cardiac tumor, diagnosis, chemotherapy, radiotherapy, immunohistochemistry, fluorescence *in situ* hybridization

## Abstract

Primary malignant cardiac tumors rarely occur, and cardiac synovial sarcoma (SS) is especially rare among such tumors. Herein, we present the case of a 35-year-old female with primary cardiac SS treated with surgery, chemotherapy, and radiotherapy. She presented with chest symptoms and underwent imaging examinations. A cardiac tumor was suspected, and an open biopsy was performed. The pathological findings suggested cardiac SS. Next, we performed a resection, and the tumors persisted at a macroscopic level. Immunohistochemistry was negative for SS18-SSX and positive for the SSX C-terminus and cytokeratin CAM5.2, a reduction of SMARCB1/INI1 was observed, and fluorescence *in situ* hybridization showed positive SS18 split staining. Owing to the FNCLCC grade 3 tumor and R2 margins, adjuvant chemotherapy with ifosfamide, doxorubicin, and radiotherapy was initiated, and the patient was diagnosed with cardiac SS. The differences in patients with cardiac SS compared with general SS include male predominance, larger tumor size, and poorer prognosis. Pathological findings of immunohistochemistry and fluorescence *in situ* hybridization were found to be more reliable than imaging findings for a correct diagnosis. Additionally, because incomplete resection is frequently performed, adjuvant therapy, including chemotherapy and radiation therapy, may be performed. The findings indicate that multiple therapies, including surgery, chemotherapy, and radiotherapy, are essential treatment strategies for improving the prognosis of patients with cardiac SS.

## 1 Introduction

Primary Cardiac tumors are generally rare, and cardiac sarcoma has an extremely low incidence rate of 0.22 per 100,000 individuals per year (1). The sites of occurrence are divided into the pericardium and the intracardium (2). Among pericardiac tumors are “primary” and “secondary” (metastatic) types, of which secondary metastatic tumors are more common, with the lungs or breasts being common primary sites (3). Malignant mesothelioma is the most common primary malignant tumor among primary pericardial malignant tumors, whereas sarcomas are extremely rare (3). In addition, pericardial tumors are characterized by a wide range of symptoms, from asymptomatic to sudden death (3). “Synovial sarcoma (SS)” is a spindle cell sarcoma that shows various degrees of epithelial differentiation and is characterized by the presence of the SS18-SSX1/2/4 fusion gene (4). Although soft tissue in the extremities is the most common site of SS occurrence, some reports of SS in other organs also exist (4). Here, we report a case of a patient with pericardiac SS who was treated with surgery, chemotherapy, and radiotherapy and compare the characteristics of cardiac SS with those of general SS in the extremities.

## 2 Case description

**Figure 1:**
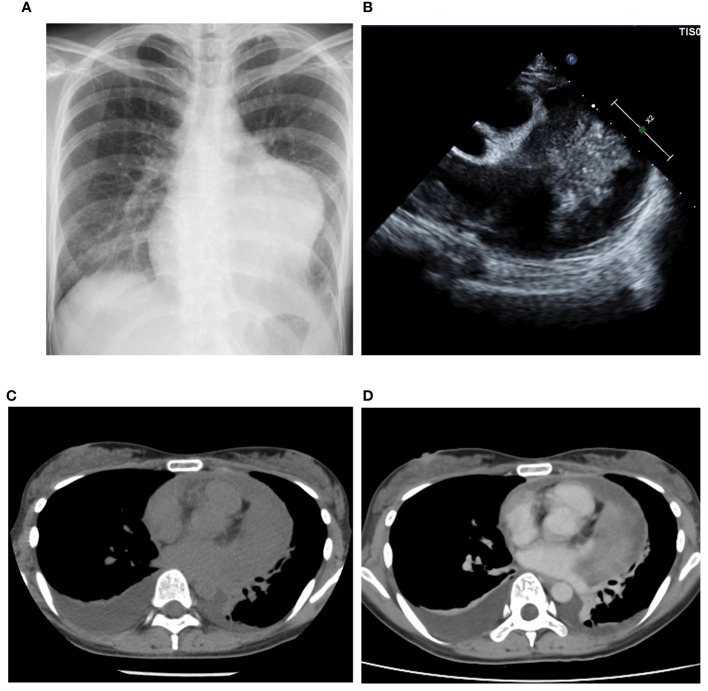
Imaging findings. Plain radiography **(A)** showing the enlargement of left second and third arc protrusion and echocardiography **(B)** showed a mass on the lateral side of the left ventricle. Computed Tomography (CT) revealed cardiac enlargement, pericardial effusion, and bilateral pleural effusion **(C)**. The enhanced CT showed tumor with faint enhancement and no calcification **(D)**.

**Figure 2:**
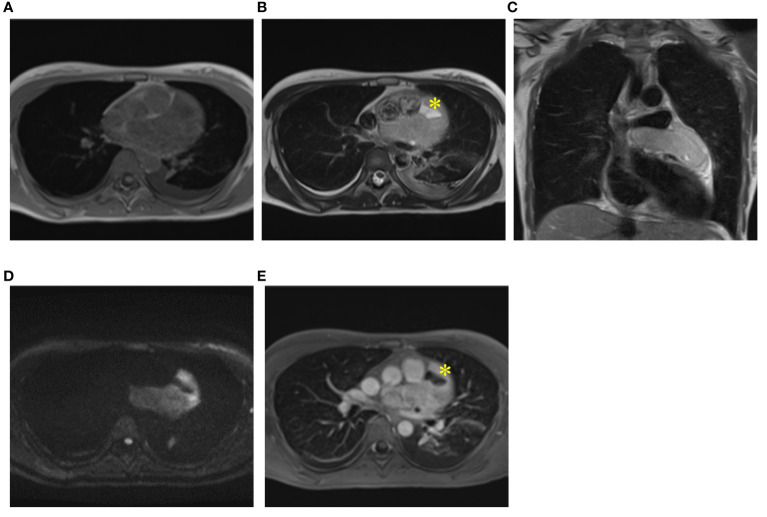
MRI findings. Magnetic Resonance Imaging (MRI) revealed a light low signal compared to muscle on T1WI **(A)**, a light high signal on T2WI with a capsule being low signal on T2WI **(B, C)** and a high signal on DWI **(D)**. Delayed enhancement was observed on gadolinium-enhanced T1-weighted images **(E)**. Cystic lesions inside of tumor (yellow asterisk) with high signals on T2WI **(B)** and without enhancement **(E)**.

A 35-year-old female presented to our hospital with fever, chest pain, and dyspnea on exertion, without any apparent cause, 1 month prior to admission. Two weeks later, she visited a physician and was diagnosed with pleural effusion, pericardial effusion, and a mass on computed tomography (CT) and echocardiography. She was admitted to another hospital after being suspected of having a sarcoma due to a thoracoscopic biopsy. Her congestive heart failure was treated with tolvaptan, and her condition improved. The patient was then referred to our hospital for tumor resection. At the time of admission, she exhibited normal pulse as 81 beats per minute, normal temperature as 36.7°C, normal oxygen saturation of 98% in room air, and normal blood pressure of 95/59 mmHg. Electrocardiography revealed normal sinus rhythm, and plain chest radiography revealed cardiomegaly in the left second and third arc protrusion (**Figure 1A**). Echocardiography revealed a mass on the lateral side of the left ventricle (LV) (**Figure 1B**). LV ejection fraction was 79%, and LV wall motion was normal; however, diastolic dysfunction was present. Although LV and left atrium (LA) appeared compressed by the tumor, the size of all four chambers was normal. There was no valve disease other than moderate mitral regurgitation without leaflet prolapse. CT revealed cardiac enlargement, a large pericardial effusion, and bilateral pleural effusion (**Figure 1C**). Enhanced CT showed uniform thickening of the pericardium and a faintly contrasting tumor that extended from the left side of the pericardial sinus to the left atrial appendage and left pulmonary vein orifice. No calcification, pericardial or thoracic dissemination or metastasis was found (**Figure 1D**). Magnetic resonance imaging (MRI) revealed a low signal compared to the muscle on T1WI (**Figure 2A**), a light-high signal on T2WI with a capsule showing a low signal (**Figures 2B, C**), and a high signal on DWI (**Figure 2D**). Delayed enhancement was observed on gadolinium-enhanced T1-weighted images (**Figure 2E**). In addition, there were cystic lesions inside the tumor with high signals on T2WI without enhancement, and a fluid level was observed inside the cystic lesion (**Figures 2B, E**).

## 3 Diagnostic assessment

**Figure 3:**
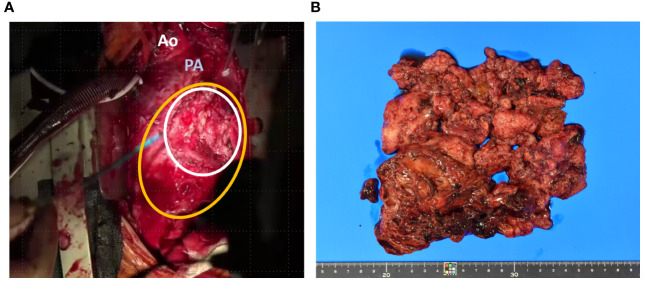
Intraoperative and pathological findings of the resected specimen. the tumor (white line) had infiltrated the myocardium on left ventricle (yellow line) **(A)**. Macroscopically, the tumor measured 12.5 × 9 × 3 cm with a grayish white color and bleeding **(B)**. Ao; Aorta, PA; Pulmonary artery.

**Figure 4:**
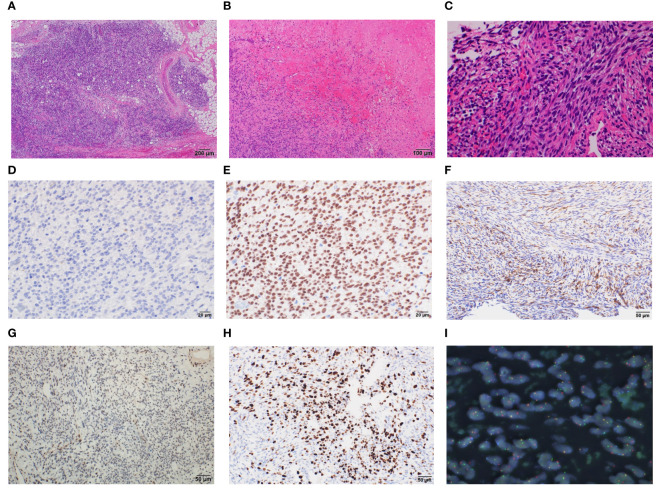
Histopathological findings of cardiac monophasic synovial sarcoma. Resected specimens demonstrate that the tumor has spindle cell components with mitotic activity, focal necrosis, no rhabdoid cells **(A–C)**. Immunohistochemical results for the indicated proteins are shown: **(D)** SS18::SSX; **(E)** SSX; **(F)** CAM5.2; **(G)** SMARCB1/INI1; **(H)** Ki67, magnification ×400 **(D, E)**, ×200 **(F–H)**. Fluorescence *in situ* hybridization showing SS18 split signal **(I)**.

Biopsy suggested pericardial SS, and taken together, we diagnosed the patient with pericardial SS (T3, N0, and M0) and planned tumor resection. Surgery was performed through median sternotomy without cardiac arrest and using cardiopulmonary bypass, and a tumor in the pericardial sac was confirmed after cutting the cardiac membrane. The adhesions were particularly strong in the LV and aorta; therefore, we removed the entire mass and instead performed piece-by-piece removal. The tumor extended to the left coronary artery, particularly at the proximal side of the left circumflex branch, where the demarcation with the tumor was indistinct. The tumor was excised with careful avoidance of injury to the myocardium and coronary arteries, thus obviating the need for LV reconstruction (**Figure 3A**). Macroscopically, the tumor measured 12.5 × 9 × 3 cm and had a grayish white color with bleeding (**Figure 3B**). Histopathologically, the tumor was composed of spindle cells, showing a monophasic pattern with mitotic activity (score 3), focal necrosis (score 1), no rhabdoid cells, and a staghorn-shaped vascular structure (**Figures 4A–C**). The results of immunohistochemistry (IHC) were as follows; SS18-SSX (−) (**Figure 4D**), SSX C-terminus (+) (**Figure 4E**), cytokeratin CAM5.2 (+, focal) (**Figure 4F**), AE1/AE3 (+, focal) (data not shown), EMA (−), calretinin (+, focal), SMARCB1/INI1(weak or loss) (**Figure 4G**). The Ki67 (MIB-1) labeling index was 60% (**Figure 4H**). Fluorescence *in situ* hybridization (FISH) showed a SS18 split signal in 71 (71%) of 100 tumor cells (**Figure 4I**). The final diagnosis of primary cardiac SS was made based on the IHC, FISH, and imaging findings, and the tumor was classified as Grade 3 according to the FNCLCC Grading System. Because the surgical margin was evaluated as R2 and the tumor was high-grade, we administered adjuvant chemotherapy with ifosfamide and doxorubicin in six cycles, followed by radiotherapy with 45 Gy in 25 fractions. The patient was free of recurrence and metastasis 1 year after surgery. The postoperative echocardiogram showed that LV contractility and the degree of mitral regurgitation remained the same as preoperative values; however, left atrial and LV decompensation was relieved, and LV diastolic dysfunction improved. Her original symptoms comprised fever and dyspnea; the latter improved after diuretics were started preoperatively, and there was no postoperative dyspnea.

## 4 Discussion

Synovial sarcoma (SS) is a relatively rare type of sarcoma, accounting for approximately 5–10% of all sarcomas (5). The most common site of occurrence is the extremities, accounting for 70% of incidences (4), whereas SS rarely occurs in the kidneys, lungs, stomach, bones, or heart (4, 6). Because cardiac SS occurs extremely rarely, we selected three retrospective case reviews that included adequate clinical information (2, 7, 8). Based on these reviews, we summarized the clinical features, pathological findings, imaging findings, and treatment of cardiac SS compared to those of general SS (almost the same as SS in the extremities).

The average age of patients with cardiac SS is three decades and there is a male predominance, with a ratio of 3–4:1 (2, 7, 8). The average age of patients with general SS is 34 years, with approximately 77% of cases occurring before the age of 50 years and a slight male predominance suggesting the sex difference was relatively characteristic (4). The average tumor size of cardiac SS is 9.75 cm, which is larger than the mean tumor size of general SS (6 cm), and cardiac SS has been reported in 91% of patients with SS tumors > 5 cm and in 32% of patients with SS tumors > 10 cm (2, 7, 8). In this patient, similar to previous reports, the tumor measured 12 cm and was considered large. Although a correlation exists between tumor size and prognosis in patients with general SS (9), there is no such correlation in patients with cardiac SS (2, 8). This discrepancy may be due to the larger size and poor prognosis of patients with cardiac SS. The clinical symptoms of cardiopulmonary diseases include respiratory distress, chest pain, and coughing (2); however, nonspecific symptoms such as fever, fatigue, syncope, cardiac tamponade, and electrocardiographic abnormalities, including right bundle branch block, axis deviation, and total AV, have also been reported (2).

Echocardiography is useful for discriminating between the presence and absence of cardiac tumors (2). The usefulness of ultrasound in soft tissue tumors of the extremities, as well as in cardiac tumors, has been reported; however, MRI and biopsy are needed to estimate the precise differential diagnosis (10). Calcification was observed in approximately 30–40% of patients on plain radiography and CT, and the presence of calcification was associated with a better prognosis (5, 11). In MRI, the findings of general SS include the triple, pseudo-cystic, and tail signs; however, the positive rates of these signs are relatively low at 43%, 22%, and 43%, respectively (11). Although pseudocysts were observed in the present patient, it was not realistic to diagnose SS based on these findings. In addition, the tail sign on MRI, which is a representative feature of infiltrative capability, is often observed in sarcomas, including SS. In this patient, no tail signs were observed on MRI; however, caution is required because infiltration into the myocardium was observed macroscopically during surgery. The correlation between the triple sign and tail signs on MRI and prognosis is currently controversial (11, 12). Patients with cardiac SS with suspected pericarditis on echocardiography and MRI have also been reported (13). Based on these findings, diagnosis using imaging alone may be limited, and the role of pathological findings is important for a definitive diagnosis.

SS is pathologically classified into three types: monophasic, biphasic, and poorly differentiated (4). Monophasic accounts for 60-70% of general SS (4), and monophasic SS accounts for 50–60% of cardiac SS, suggesting that the histological types in both general and cardiac SS display similar trends (2, 7, 8). Nor of the correlation between the pathological classification and the prognosis of patients with cardiac SS. In general, the FNCLCC grade, but not histological type, correlates with prognosis (5). IHC is important for the precise diagnosis of SS, and the positivity rates of cytokeratin, EMA, bcl-2, and CD99 are generally reported to be high (5). This trend was also observed in cardiac SS (2). Although IHC has limitations due to the overlap between SS and other sarcomas, the development of SS18-SSX antibodies and SSX C-terminal antibodies in IHC could lead to innovation in accurate IHC diagnosis because of their high sensitivity and specificity (14). In addition, the usefulness of the reduction in SMARCB1/INI1 and the expression of TLE-1 has also been reported (5). Although IHC has been developed, it is still necessary to perform molecular confirmation using FISH or PCR. The sensitivity of the SS18 FISH assay is approximately 90%, although decalcification may dampen sensitivity by up to 36% (15, 16). Yoshida et al. reported FISH-negative patients with SS, among whom SS was diagnosed via IHC using SS18-SSX antibody, SSX C-terminus antibody, SMARCB1/INI1, FISH, and RNA-sequencing (15). In pericardial SS, mesothelioma, especially sarcomatoid mesothelioma, is considered the most important in differential diagnosis (17). The pathological characteristics of mesothelioma, including the loss of BAP1 in IHC and the deletion of p16 (CDKN2A) in FISH, have been reported (17).

The standard treatment for SS is surgery with complete resection, whereas chemotherapy and radiotherapy are performed in patients with incomplete resection (9). Regarding the characteristics of surgery in patients with cardiac SS, there have been many cases of incomplete resection, including R1 and R2 (2, 7, 8). Our patient also underwent R2 resection because of infiltration into the myocardium, and there is a possibility that there have been many patients with tumor adhesion and infiltration. In general, complete resection of the tumor results in a better prognosis (9); however, the correlation between complete resection and prognosis in cardiac SS remains controversial (2, 7, 8). Owing to the limitations of surgery in cardiac SS, chemotherapy and radiotherapy may play a more significant role in cardiac SS than in general SS. The improvement of overall survival with chemotherapy and/or radiotherapy in cardiac SS showed the same trend as that in general SS (2, 8, 9). In chemotherapy for general SS, the efficacy of the combination of ifosfamide and Doxorubicin is higher than that of ifosfamide or Doxorubicin alone (18). Based on these findings, we selected this regimen. Although the effect of radiation therapy on improving prognosis is controversial, it contributes to a reduction in local recurrence (9, 19). Therefore, radiotherapy is thought to have great advantages in cardiac SS, because there have been many cases of incomplete resection. Radiation therapy for cardiac sarcoma, including Cyberknife-based stereotactic radiation and carbon ion radiation, has also been reported (20, 21). The survival rate in cardiac SS is approximately 60% at 1 year and 30% at 5 years, which is significantly lower than the 5-year survival rate of 79% in general SS (2, 9). Considering these factors, surgery, chemotherapy, and radiation therapy are essential and have been proposed as standard regimens for the treatment of cardiac SS.

In conclusion, we report an extremely rare case of pericardial SS. Considering previous reports, the characteristics of cardiac SS are i) larger size, ii) incomplete resection during surgery, and iii) worse prognosis. Therefore, multidisciplinary treatment with surgery, chemotherapy, and radiation is the only and best way to improve patient prognosis.

## Data availability statement

The raw data supporting the conclusions of this article will be made available by the authors, without undue reservation.

## Ethics statement

Ethical approval was not required for the studies involving humans because written informed consent from patients was obtained. Written informed consent was obtained from the individual(s) for the publication of any potentially identifiable images or data included in this article.

## Author contributions

TK: Conceptualization, Data curation, Funding acquisition, Investigation, Writing – original draft, Writing – review & editing. TN: Conceptualization, Writing – original draft, Writing – review & editing, Data curation. TT: Conceptualization, Investigation, Writing – original draft, Writing – review & editing. KO: Investigation, Writing – original draft, Writing – review & editing. KI: Writing – original draft, Writing – review & editing. SK: Writing – original draft, Writing – review & editing. CM: Writing – original draft, Writing – review & editing. RT: Writing – original draft, Writing – review & editing. TJ: Writing – original draft, Writing – review & editing. JI: Conceptualization, Investigation, Writing – original draft, Writing – review & editing.
